# Laparoscopic sleeve gastrectomy alters ^1^H-NMR-measured lipoprotein and glycoprotein profile in patients with severe obesity and nonalcoholic fatty liver disease

**DOI:** 10.1038/s41598-020-79485-7

**Published:** 2021-01-14

**Authors:** Noemí Cabré, Míriam Gil, Núria Amigó, Fedra Luciano-Mateo, Gerard Baiges-Gaya, Salvador Fernández-Arroyo, Elisabet Rodríguez-Tomàs, Anna Hernández-Aguilera, Helena Castañé, Marta París, Fàtima Sabench, Daniel Del Castillo, Jordi Camps, Jorge Joven

**Affiliations:** 1 Department of Medicine and Surgery, Universitat Rovira i Virgili, C. Sant Llorenç, 21, 43201 Reus, Spain; 2 Unitat de Recerca Biomèdica (URB-CRB), Hospital Universitari de Sant Joan, Institut d’Investigacio Sanitaria Pere Virgili, Universitat Rovira i Virgili, C. Sant Joan S/N, 43201 Reus, Tarragona Spain; 3 Biosfer Teslab, Universitat Rovira i Virgili, Av. Universitat 1, 43204 Reus, Tarragona Spain; 4 Department of Surgery, Hospital Universitari de Sant Joan, Institut d’Investigació Sanitaria Pere Virgili, Universitat Rovira i Virgili, Av. Doctor Josep Laporte 2, 43204 Reus, Tarragona Spain; 5 The Campus of International Excellence Southern Catalonia, Tarragona, Spain

**Keywords:** Medical research, Glycobiology, Lipidomics, Lipids, Gastrointestinal diseases, Nutrition disorders

## Abstract

Patients with morbid obesity frequently present non-alcoholic fatty liver (NAFL) and non-alcoholic steatohepatitis (NASH) associated with pro-atherogenic alterations. Laparoscopic sleeve gastrectomy (LSG) is an effective treatment for weight reduction, and for the remission of hepatic alterations. Using ^1^H-nuclear magnetic resonance (^1^H-NMR), we investigated the effects of LSG on lipoprotein and glycoprotein profile in patients with morbid obesity and liver disease. We included 154 patients with morbid obesity (49 non-NASH, 54 uncertain NASH, 51 definite NASH). A blood sample was obtained before surgery and, in patients with definite NASH, one year after surgery. Patients with NASH had increased concentrations of medium and small VLDL particles, VLDL and IDL cholesterol concentrations, IDL, LDL, and HDL triglyceride concentrations, and elevated glycoprotein levels. These changes were more marked in patients with type 2 diabetes mellitus. LSG produced significant decreases in the concentration of VLDL particles, VLDL cholesterol and triglycerides, an increase in the concentration LDL particles and LDL cholesterol concentrations, and a decrease in protein glycation. We conclude that patients with obesity and NASH had significant alterations in circulating levels of lipoproteins and glycoproteins that were associated with the severity of the disease. Most of these changes were reversed post-LSG.

## Introduction

The prevalence of obesity has increased steadily in recent decades such that it has reached epidemic proportions. Subjects with morbid obesity share a common metabolic background, and their clinical status is frequently influenced by similar environmental factors such as an unbalanced diet or a sedentary lifestyle^1,2^. Non-alcoholic fatty liver disease (NAFLD) is an important comorbidity linked to obesity. This disorder includes a broad spectrum of lesions that can range from benign simple steatosis (nonalcoholic fatty liver, NAFL) to the more severe nonalcoholic steatohepatitis (NASH), which can evolve to fibrosis, cirrhosis, liver failure, and hepatocarcinoma^3^.

Fatty liver is associated with increased concentrations of the circulating levels of low-density lipoprotein (LDL) cholesterol and triglycerides, together with decreased high-density lipoproteins (HDL) concentrations resulting in an increased risk of cardiovascular disease, in diabetic and non-diabetic patients^4–6^. Until recently it has not been possible to investigate in depth the alterations in lipoprotein metabolism associated with NASH, since the conventional lipoprotein panel does not provide sufficient information on the subtle changes that can occur in these patients^7,8^. Fortunately, there have been significant technological advances in recent years in the rapid, reliable and detailed analyses of circulating lipoproteins. ^1^H-nuclear magnetic resonance (^1^H-NMR) has the advantage of simultaneously quantifying the number, size and composition of lipoprotein particles, thus providing a better understanding of modifications associated with metabolic disturbances such as NAFLD^9^. In addition, ^1^H-NMR spectroscopy allows measurement of different classes of glycoproteins, and this is clinically relevant in patients because increased protein glycation is a marker of the extent of type 2 diabetes mellitus. Also, increased plasma glycoprotein concentrations have been associated with insulin resistance and obesity^10,11^.

Laparoscopic sleeve gastrectomy (LSG) is a widely-used surgical procedure for the treatment of morbid obesity and its associated comorbidities^12^. Patients who benefit from LSG not only reduce weight, but also improve insulin resistance and histological features of NAFLD^13,14^. For example, recent studies have reported that patients with morbid obesity treated with LSG have significant decreases in liver volume and hepatic steatosis at 6 months^15–17^ and improvement of other serious comorbidities, including but not limited to type 2 diabetes, hypertension, and obstructive sleep apnea^18^. A study by our research group showed that the improvement in the histology and liver function of these patients after LSG was associated with mechanisms that involve the reduction of oxidative stress and inflammatory processes^3^. Hence, the present study investigated the effect of LSG on ^1^H-NMR-lipoprotein and glycoprotein profile in patients with morbid obesity, and with several degrees of hepatic alterations.

## Methods

### Study design and participants

This post hoc retrospective cohort study includes new objectives derived from a previous prospective longitudinal study investigating the molecular mechanisms associated with liver injury in morbid obesity and searching for plasma biomarkers of obesity-associated liver disease^3,19^. In this previous prospective study, we included 436 patients with severe obesity (body mass index, BMI > 40 kg/m^2^) who underwent LSG. All subjects provided 12-h fasting blood samples immediately before surgery together with an intraoperative wedge-liver biopsy. Patients that were diagnosed as having definite NASH were asked to have a second blood extraction and an additional liver biopsy at 12 months post-surgery^3^. In the present study we selected 154 of those patients who were matched for age, sex, BMI, and incidence of diabetes mellitus, hypertension and dyslipidemia, and who were representative of the three most frequent degrees of liver injury: Patients with mild hepatic lesions without NASH (n = 49), patients with uncertain NASH (n = 54), and patients with definite NASH (n = 51). Samples were stored at − 80 °C in the Biobank of our Institution *(Banc de Mostres Biològiques, Institut d’Investigació Sanitària Pere Virgili)*. Clinical indication for LSG was according to guidelines currently used in pre-operative evaluation^20^. We excluded patients with current, or past, history of daily alcohol abuse (≥ 30 g for men and ≥ 20 g for women), long-term consumption of hepatotoxic drugs, and liver disease of infectious origin. LSG was performed under general anesthesia with the patient in the Lloyd-Davies position. A five-port technique was used in all patients. The greater gastric curvature was dissected, separated from the gastroepiploic arcade of the greater omentum, and continued to the His angle. The gastric transection was performed under the guidance of a 38-Fr Faucher bougie. Three cm was the distance from the pylorus to the first section point (measured intraoperatively with a ribbon). The suture line was reinforced using polycarbonate derivatives of polyglycolic acid (SEAMGUARD, W.L. GORE & ASSOCIATES, USA) in order to avoid hemorrhagic processes and leaks. A methylene blue leak was always performed before closing abdominal wall^21^.

All experimental protocols were approved by the Ethics Committee (Institutional Review Board) of Hospital Universitari de Sant Joan (OBESPAD/14.07-31proj3), and patients provided fully informed, signed consent (OM-NAFLD, ESO3/18012013 project). All methods were carried out in accordance with relevant guidelines and regulations.

### Histological analysis

Liver biopsies were obtained from the same site in all patients, and were examined by a pathologist blinded to clinical data. Samples were processed conventionally for diagnostic purposes and histological grading and staging, as described^22^. Steatosis was graded in four categories, depending on whether fat droplets occupied < 5%, 6–33%, 34–66%, or ≥ 67% of the total microscopic field; inflammation was graded as: no foci of lobular inflammation observed, or < 2, 2–4, and > 4 foci per field; fibrosis was classified as: absence of fibrosis (Stage 0), mild to moderate fibrosis (Stages 1 and 2), and bridging fibrosis (Stage 3). The presence or absence of NASH was estimated using the NAFLD activity score (NAS score) and defined as the sum of steatosis, inflammation and hepatocyte ballooning. Patients were classified in three categories: non-NASH (n = 49; NAS ≤ 2), uncertain NASH (n = 54; NAS 3–4) and definite NASH (n = 51; NAS ≥ 5)^23^.

### Lipoprotein and glycoprotein analyses by ^1^H-NMR spectroscopy

Whole blood was centrifuged at 2500×*g* and 4 °C and serum was aliquoted and stored at − 80 °C until analyses were performed. Lipoproteins and glycoproteins were analyzed by the ^1^H-NMR-based LIPOSCALE test, as previously reported^9–11^. Cholesterol and triglyceride concentrations, particle size and concentration of the four main classes of lipoproteins (very-low-density lipoproteins or VLDL, low-density lipoproteins or LDL, intermediate-density lipoprotein or IDL, and high-density lipoproteins or HDL), as well as particle concentration of nine lipoprotein subclasses (large, medium and small VLDL, LDL, and HDL) were analyzed. The methyl signals of the serum 2D ^1^H-NMR spectra were derived from deconvolution analysis using 9 lorentzian functions to determine the lipid concentration of each lipoprotein, and its diffusion coefficient (Z); these are estimations of particle diameter. Finally, information on lipid concentrations and particle volumes derived from the diffusion coefficients were combined to quantify the number of lipoprotein particles required to transport the measured lipid concentration of each lipoprotein subclass.

Glycoproteins were analyzed at the 2.15–1.90 ppm region of the ^1^H-NMR spectrum i.e. where the glycoproteins resonate. We determined the total area (proportional to concentration), height (H), position and bandwidth (W). The GlycA area provided values for the concentration of acetyl groups of protein-bound *N*-acetylglucosamine and *N*-acetylgalactosamine. The GlycB area measured N- acetylneuraminic acid while the GlycF area measured the concentrations of the acetyl groups of *N*-acetylglucosamine, *N*-acetylgalactosamine and *N*-acetylneuraminic acid not bound to proteins (unbound, free fraction). H/W ratios of GlycA and GlycB (a parameter associated with the aggregation state of the sugar–protein bonds) were calculated. Height was measured as the difference from baseline to maximum of the corresponding ^1^H-NMR peaks. Width values correspond to the peak width at half height. The areas termed low molecular weight molecules (LMWM) 1 and 2 mostly correspond to glutamine and glutamate, although interference from other metabolites can occur^9–11^.

### Standard biochemical tests

Serum cholesterol, high-density lipoprotein (HDL) cholesterol, serum triglycerides, glucose, albumin, and insulin concentrations were analyzed by conventional methods in a Roche Modular Analytics P800 system (ROCHE DIAGNOSTICS, Basel, Switzerland).

### Statistical analysis

The Kolmogorov–Smirnov test was used to assess the normality distribution of the variables. Wilcoxon rank-sum test or Kruskal–Wallis test (non-parametric) were used to compare independent quantitative variables, and the—square test was used to compare categorical variables. Wilcoxon signed-rank test was employed to compare dependent variables. Correlations between quantitative variables were analyzed with Spearman’s Rho test. Multivariate analysis was applied to pattern recognition, including the supervised partial least squares discriminant analysis (PLS-DA). The relative magnitude of observed changes was evaluated using the variable importance in projection (VIP) score^24^. Statistical significance was set at p ≤ 0.05. Statistical analyses were performed with the SPSS 22.0 package and the R program version 3.4. METABOANALYST 4.0 program (available on the web http://www.metaboanalyst.ca/) was used to generate scores and loading plots.

## Results

### Patient characteristics

Serum aminotransferase activities and the HOMA-IR index were higher in patients with definite NASH compared to those without NASH. We did not find any significant differences in age, sex, BMI, incidence of type 2 diabetes mellitus, hypertension, dyslipidemia, or lipid profile as analyzed by standard clinical laboratory tests (Table 1). However, the alterations observed were associated with the highest degrees of steatosis and fibrosis, but not with lobular inflammation (Supplementary Table S1).

**Table 1:** Clinical, biochemical, and histological variables segregated with respect to the NAS score.

	Non-NASH (n = 49)	Uncertain NASH (n = 54)	Definite NASH (n = 51)	*P* value
**Clinical characteristics**				
Female, n (%)	39 (79.6)	39 (72.2)	40 (78.4)	0.634
Age, years	48.0 (40.5–62.5)	55.5 (48.0–61.0)	48.0 (43.0–60.0)	0.119
BMI, Kg/m^2^	47.0 (42.9–52.6)	49.0 (45.8–54.1)	49.5 (43.7–54.3)	0.236
T2DM, n (%)	18 (36.7)	26 (48.1)	26 (51.0)	0.321
Arterial hypertension, n (%)	34 (69.4)	36 (66.7)	35 (68.6)	0.954
Dyslipidemia, n (%)	19 (38.8)	25 (46.3)	25 (49.0)	0.569
**Medication (%)**				
Metformin	9 (18.4)	20 (37.0)	22 (43.1)	0.023
Insulin	3 (6.1)	7 (13.0)	3 (5.9)	0.335
Sulfonylureas	4 (8.2)	6 (11.1)	5 (9.8)	0.881
ACEIs + ARA-II	22 (44.9)	26 (48.1)	25 (49.0)	0.910
Diuretics	7 (14.3)	9 (16.7)	9 (17.6)	0.897
Statins	14 (28.6)	17 (31.5)	13 (25.5)	0.827
**Biochemical variables**				
Total cholesterol, mmol/L	4.5 (3.6–5.3)	4.6 (3.8–5.3)	4.7 (4.1–5.3)	0.535
HDL cholesterol, mmol/L	1.2 (0.8–1.5)	1.2 (0.8–1.6)	1.0 (0.9–1.3)	0.592
LDL cholesterol, mmol/L	2.6 (1.8–3.1)	2.5 (1.9–3.2)	2.7 (2.2–3.3)	0.303
Triglycerides, mmol/L	1.5 (1.2–2.2)	1.7 (1.3–2.6)	1.9 (1.4–2.5)	0.199
Glucose, mmol/L	7.1 (6.0–8.6)	7.9 (6.1–10.1)	8.1 (6.5–10.9)^a^	0.114
Insulin, pmol/L	80.5 (36.8–140.3)	89.6 (41.8–172.5)	93.9 (60.9–146.4)	0.383
HOMA-IR	3.8 (1.7–6.9)	4.7 (2.1–11.4)	5.3 (3.3–9.0)^a^	0.090
AST, U/L	30 (18–42)	30 (24–48)	48 (30–78) ^a,b^	< 0.001
ALT, U/L	30 (18–48)	36 (18–48)	54 (36–84)^a,b^	< 0.001
GGT, U/L	24 (18–30)	24 (12–36)	30 (18–72)^a,b^	0.004
**Steatosis, n (%)**				
< 5%	24 (49.0)	8 (14.8)	–	
5–33%	23 (46.9)	31 (57.4)	4 (7.8)	< 0.001
34–66%	2 (4.1)	14 (25.9)	26 (51.0)	
> 66%	–	1 (1.9)	21 (41.2)	
**Lobular inflammation, n (%)**				
No foci	19 (38.8)	4 (7.4)	–	
< 2 foci per 200 × field	27 (55.1)	20 (37.0)	10 (19.6)	< 0.001
2–4 foci per 200 × field	3 (6.1)	23 (42.6)	28 (54.9)	
> 4 foci per 200 × field	–	7 (13.0)	13 (25.5)	
**Hepatocellular ballooning, n (%)**				
None	39 (79.6)	24 (44.4)	6 (11.8)	< 0.001
Few cells	10 (20.4)	28 (51.9)	29 (56.9)	
Many cells	–	2 (3.7)	16 (31.4)	
**Fibrosis, n (%)**				
None (Stage 0)	18 (36.7)	11 (20.4)	12 (23.5)	0.003
Perisinusoidal or periportal (Stage 1)	17 (34.7)	26 (48.1)	10 (19.6)	
Perisinusoidal and portal (Stage 2)	11 (22.4)	14 (25.9)	15 (29.4)	
Bridging fibrosis (Stage 3)	3 (6.1)	3 (5.5)	14 (27.4)	

Values are shown as number of cases and percentages, or medians and interquartile ranges.

*ACEIs* angiotensin-converting-enzyme inhibitor; *ALT* alanine aminotransferase; *AST* aspartate aminotransferase; *ARA-II* angiotensin II receptor antagonists; *BMI* body mass index; *GGT* γ-glytamyl transferase; *HOMA-IR* homeostatic model assessment of insulin resistance; *HDL* high-density lipoproteins; *LDL* low-density lipoproteins; *NAS* nonalcoholic fatty liver disease activity score; *NASH* nonalcoholic steatohepatitis; *T2DM* type 2 diabetes mellitus. Superscript letters indicate significant (at least p < 0.05) differences between: ^a^ non-NASH *vs.* definite NASH; ^b^uncertain NASH *vs.* definite NASH; *Global *P* value using the Kruskal–Wallis one-way analysis of variance.

### Hepatic alterations and type 2 diabetes mellitus influences serum ^1^H-NMR lipoprotein and glycoprotein profiles

**Figure 1:**
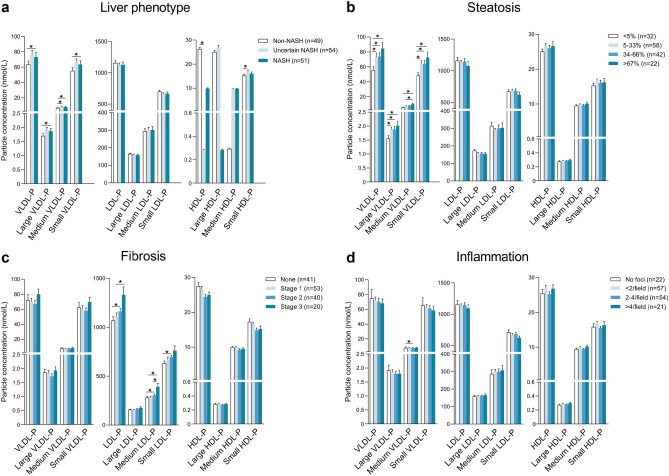
Concentration of particles of each subclass of lipoproteins in patients with morbid obesity pre-surgery. Patients were segregated according to the severity of the liver injury (**a**), percentage of steatosis (**b**), degree of fibrosis (**c**), and inflammation (**d**). Results are shown as a means and standard errors. Asterisks denote significance (*p ≤ 0.05) by Wilcoxon rank-sum test. HDL: high-density lipoproteins; IDL: intermediate-density lipoproteins; LDL: low-density lipoprotein; P: Particle concentration; VLDL: very-low-density lipoproteins.

Patients with definite NASH had significantly higher values of medium and small VLDL particles, VLDL and IDL cholesterol concentrations, and IDL, LDL, and HDL triglycerides than those with uncertain NASH. Glycoproteins were significantly elevated in the LMWM2 area (Fig. 1a and Table 2). Also, patients with the most severe degrees of steatosis had significantly higher values of medium and small VLDL particles, VLDL and IDL cholesterol concentrations, triglycerides in all lipoprotein fractions, and LMWM2 area than patients with less severe steatosis (Fig. 1b and Supplementary Table S2). Patients with advanced fibrosis had significantly elevated values of medium and small LDL particles, IDL, LDL, HDL cholesterol concentrations, LDL triglycerides, and LMWM1 area that patients with less severe steatosis (Fig. 1c and Supplementary Table S3). Patients with more inflammation had significantly higher medium VLDL particles and HDL triglyceride concentrations (Fig. 1d and Supplementary Table S4).

**Table 2:** Serum cholesterol and triglyceride concentrations in lipoprotein fractions, lipoprotein particle diameter and glycoprotein variables measured by ^1^H-NMR segregated with respect to the NAS score.

	Non-NASH (n = 49)	Uncertain NASH (n = 54)	Definite NASH (n = 51)	*P* value*
**Cholesterol, mg/dL**				
VLDL	18.1 (14.4–27.0)	24.1 (18.2–32.1)^a^	24.9 (19.0–37.6)^b^	0.013
IDL	9.4 (7.5–11.0)	10.7 (9.1–14.3)^a^	12.1 (9.9–15.9)^b^	< 0.001
LDL	108.5 (92.5–126.5)	106.9 (91.9–122.2)	103.7 (84.2–121.9)	0.721
HDL	44.9 (38.2–51.9)	48.1 (37.9–53.1)	44.1 (39.7–51.3)	0.784
**Triglycerides, mg/dL**				
VLDL	73.5 (53.3–94.8)	85.2 (60.5–113.4)	95.9 (58.2–128.8)^b^	0.056
IDL	10.1 (8.5–11.4)	11.9 (10.1–14.6)^a^	12.5 (10.1–16.0)^b^	< 0.001
LDL	14.6 (11.1–16.7)	16.4 (13.0–20.4)^a^	16.7 (12.4–21.9)^b^	0.024
HDL	18.0 (13.7–22.0)	22.3 (16.9–29.6)^a^	21.0 (18.1–32.3)^b^	0.002
**Lipoprotein particle diameter, Z**				
VLDL	42.0 (41.8–42.3)	42.0 (41.8–42.3)	42.0 (41.8–42.3)	0.904
LDL	21.0 (20.6–21.1)	21.0 (20.7–21.2)	21.0 (20.7–21.2)	0.755
HDL	8.3 (8.2–8.4)	8.3 (8.2–8.4)	8.3 (8.2–8.4)	0.276
**Glycoprotein variables**				
LMWM1 area	0.27 (0.20–0.33)	0.28 (0.16–0.40)	0.29 (0.17–0.36)	0.914
LMWM2 area	2.1 (1.6–2.6)	2.3 (1.8–2.9)^a^	2.4 (1.9–3.0)^b^	0.025
Glyc-A area	5.6 (5.0–6.3)	6.1 (5.1–7.3)	6.1 (5.0–7.1)	0.214
Glyc-B area	2.2 (1.9–2.6)	2.3 (1.9–2.7)	2.1 (1.8–2.7)	0.584
Glyc-F area	2.3 (2.0–2.6)	2.5 (2.2–2.8)^a^	2.5 (1.9–2.9)	0.047
Glyc-A width	17.2 (16.9–17.7)	17.0 (16.6–17.5)	17.5 (16.8–18.0)^c^	0.089
Glyc-B width	20.3 (18.9–21.5)	19.9 (18.6–21.4)	20.0 (18.9–20.7)	0.702
Height/Width Glyc-A	17.6 (15.8–19.5)	18.7 (16.7–21.1)^a^	18.2 (15.3–21.0)	0.137
Height/Width Glyc-B	4.8 (4.4–5.6)	5.3 (4.6–5.8)	4.9 (4.4–5.7)	0.170

Values are shown as medians and interquartile ranges.

*HDL* high-density lipoproteins; *IDL* intermediate-density lipoproteins; *LMWM* low molecular weight molecules; *LDL* low-density lipoproteins; *NAS* nonalcoholic fatty liver disease activity score; *NASH* non-alcoholic steatohepatitis; *VLDL* very low-density lipoproteins. Superscript letters indicate significant (at least p < 0.05) differences between: ^a^non-NASH *vs.* uncertain NASH, ^b^non-NASH *vs.* definite NASH and ^c^uncertain NASH *vs.* definite NASH.

*Global *P* value using the Kruskal–Wallis one-way analysis of variance.

Type 2 diabetes mellitus was associated with elevated VLDL and IDL cholesterol concentrations, VLDL, IDL and HDL triglyceride concentrations, LMWM2 and Glyc-A areas (Supplementary Table S5).

### LSG modifies serum ^1^H-NMR lipoprotein and glycoprotein profiles

**Figure 2:**
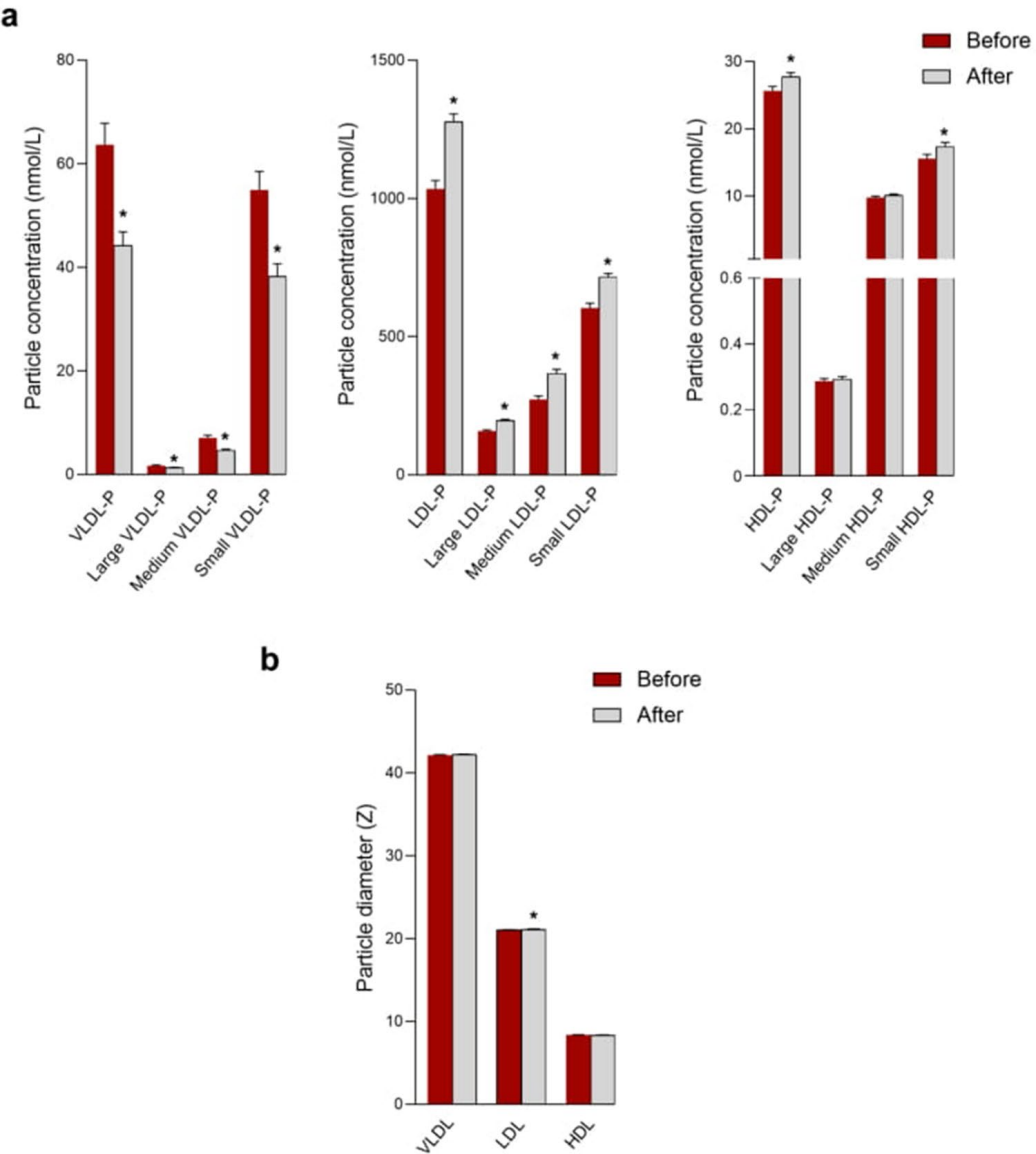
Concentration and diameter of particles of each subclass of lipoproteins pre- and post-surgery (n = 51). Results are shown as means and standard errors. Asterisks denote significance (*p ≤ 0.05) by Wilcoxon signed-rank test. *HDL* high-density lipoproteins; *IDL* intermediate-density lipoproteins; *LDL* low-density lipoproteins; *LSG* laparoscopic sleeve gastrectomy; *P* particle concentration; *VLDL* very-low-density lipoproteins; *Z* particle diameter.

One year after LSG, patients showed a significant decrease in BMI, insulin resistance and aminotransferase activities. The ^1^H-NMR lipoprotein profile showed that these patients had: a significant decrease in the concentration of small, medium and large VLDL particles, and decreased VLDL cholesterol and triglyceride concentrations; an increase of small, medium and large LDL particles and LDL cholesterol concentrations; an increase in small HDL particles and cholesterol concentrations; an increase in LDL particle diameter (Fig. 2 and Table 3). The glycoprotein profiles showed a decrease in Glyc-A, B, and F areas, Glyc-A and B width, H/W ratios, and LMWM 1 and 2 areas (Table 3).

**Table 3:** Clinical characteristic and serum cholesterol and triglyceride concentrations in lipoprotein fractions, lipoprotein particle diameter and glycoprotein variables in patients with NASH pre- and post- laparoscopic sleeve gastrectomy.

	Pre-surgery (n = 51)	Post-surgery (n = 51)	*P* value
BMI, Kg/m^2^	49.3 (44.9–55.0)	34.3 (31.4–37.1)	< 0.001
Total-cholesterol, mmol/L	5.1 (4.3–5.6)	4.8 (4.4–5.7)	0.658
HDL-cholesterol, mmol/L	1.4 (1.0–1.7)	1.5 (1.3–1.8)	0.089
LDL-cholesterol, mmol/L	2.8 (2.2–3.5)	2.8 (2.5–3.4)	0.327
Triglycerides, mmol/L	1.5 (1.1–2.3)	0.9 (0.7–1.1)	< 0.001
Glucose, mmol/L	7.5 (6.2–9.1)	4.6 (4.2–5.3)	< 0.001
Insulin, pmol/L	87.5 (47.2–183.3)	39.5 (25.6–59.7)	< 0.001
HOMA-IR	4.7 (2.4–9.0)	1.2 (0.7–1.9)	< 0.001
AST, U/L	42 (30–60)	12 (6–18)	< 0.001
ALT, U/L	36 (24–54)	18 (12–24)	< 0.001
GGT, U/L	24(12–36)	12 (6–24)	< 0.001
**Cholesterol, mg/dL**			
VLDL	21.5 (16.0–30.2)	16.0 (12.0–18.7)	< 0.001
IDL	10.3 (8.0–12.1)	9.8 (8.3–12.8)	0.683
LDL	101.3 (86.1–116.5)	126.6 (112.6–146.0)	< 0.001
HDL	43.8 (40.6–49.8)	55.1 (46.3–61.2)	< 0.001
**Triglycerides, mg/dL**			
VLDL	70.5 (55.5–101.4)	52.1 (46.3–61.2)	< 0.001
IDL	10.8 (9.0–12.9)	10.5 (8.9–12.4)	0.227
LDL	14.4 (11.5–17.8)	13.8 (11.5–17.6)	0.465
HDL	19.6 (17.3–28.6)	17.0 (14.5–19.5)	< 0.001
**Lipoprotein particle diameter, Z**			
VLDL	42.1 (41.8–42.3)	42.2 (42.0–42.4)	0.191
LDL	21.0 (20.8–21.2)	21.1 (21.0–21.3)	0.030
HDL	8.3 (8.2–8.4)	8.3 (8.2–8.4)	0.063
**Glycoprotein variables**			
LMWM1 area	0.27 (0.15–0.36)	0.15 (0.07–0.21)	< 0.001
LMWM2 area	2.2 (1.7–2.7)	1.4 (1.0–1.8)	< 0.001
Glyc-A area	5.7 (4.7–6.4)	4.3 (4.0–5.0)	< 0.001
Glyc-B area	2.2 (1.8–2.6)	1.6 (1.4–1.8)	< 0.001
Glyc-F area	2.3 (1.9–2.6)	1.7 (1.5–1.8)	< 0.001
Glyc-A width	17.0 (16.6–17.5)	17.2 (16.6–18.2)	< 0.001
Glyc-B width	20.2 (19.1–21.5)	20.2 (18.6–22.3)	< 0.001
H/W Glyc-A	17.7 (15.1–20.2)	13.4 (12.2–15.2)	< 0.001
H/W Glyc-B	5.1 (4.3–5.8)	3.8 (3.3–4.1)	< 0.001

Values are shown as medians and interquartile ranges.

*P* value calculated by the Wilcoxon signed-rank test.

*ALT* alanine aminotransferase; *AST* aspartate aminotransferase; *BMI* body mass index; *GGT* γ-glytamyl transferase; *HOMA-IR* homeostatic model assessment of insulin resistance; *HDL* high-density lipoproteins; *IDL* intermediate-density lipoproteins; *LMWM* low molecular weight molecules; *LDL* low-density lipoproteins; *VLDL* very low-density lipoproteins.

**Figure 3:**
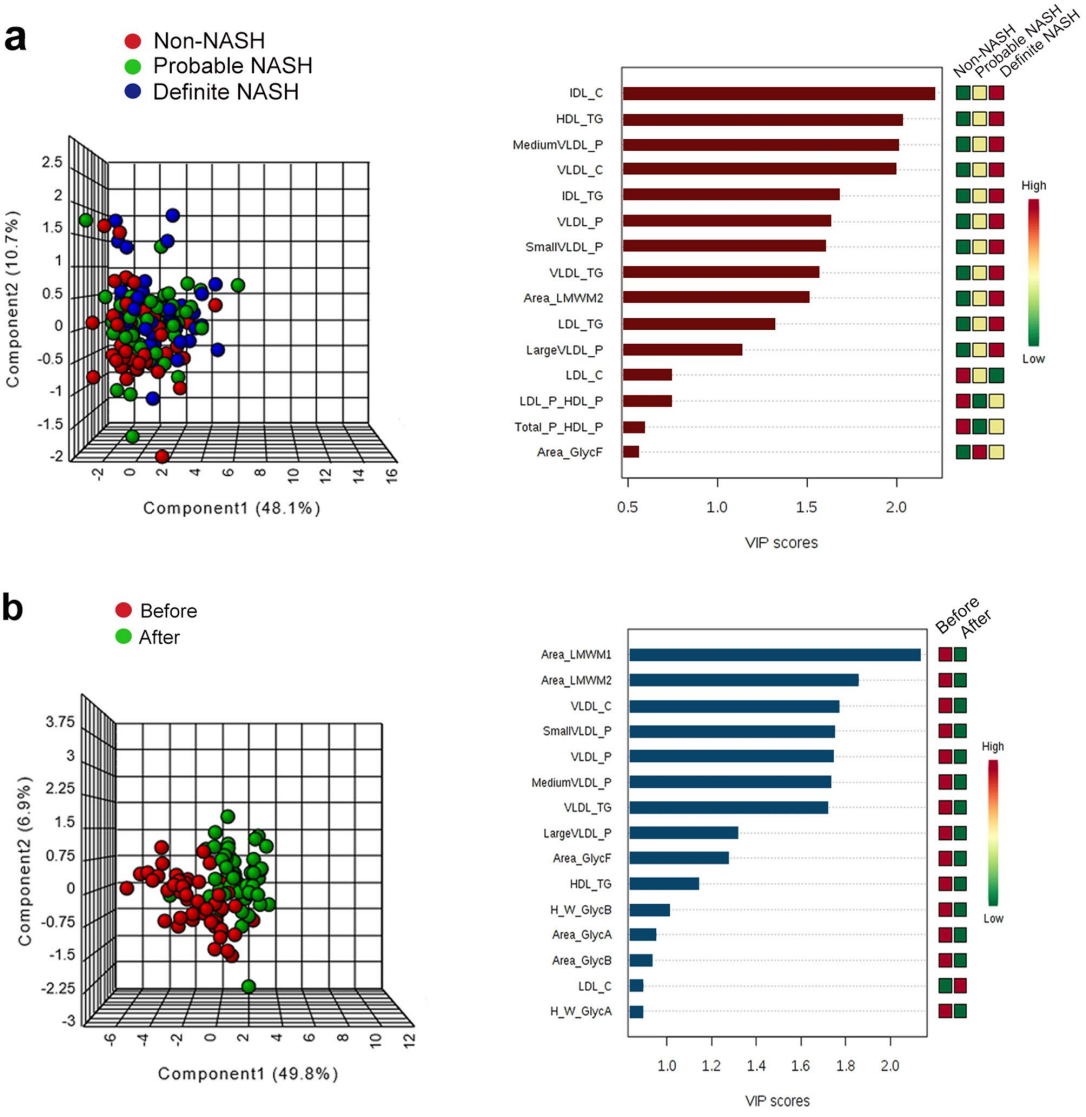
Score plots of the partial least square discriminant analysis (PLS-DA) and variable importance in projection (VIP) score analysis of measured lipoproteins and glycoproteins in patients with morbid obesity (n = 154), segregated according to the severity of hepatic alterations (**a**) or pre and post- laparoscopic sleeve gastrectomy (**b**). In the PLS-DA plots, the X and Y the axes represent combinations of the different variables analyzed so as to achieve a maximum separation between the groups. The VIP score is a measure of a variable's importance in the PLS-DA model. It summarizes the contribution a variable makes to the model. The VIP score of a variable is calculated as a weighted sum of the squared correlations between the PLS-DA components and the original variable. The weights correspond to the percentage variation explained by the PLS-DA component in the model. The number of terms in the sum depends on the number of PLS-DA components found to be significant in distinguishing the classes. *HDL* high-density lipoproteins; *IDL* intermediate-density lipoproteins; *LDL* low-density lipoprotein; *LSG* laparoscopic sleeve gastrectomy; *P* particle concentration; *VLDL* very-low-density lipoproteins; *Z* particle diameter.

The score plot of the PLS-DA analysis of pre-surgery values of serum lipoproteins and glycoproteins showed a considerable overlap such that a clear distinction between non-NASH, uncertain NASH, and NASH patient groups could not be made. To identify the lipoproteins and glycoproteins that showed the most relevant alterations, we calculated the VIP scores. This score is a measure of the variable’s degree-of-alteration associated with the disease i.e. a higher VIP score is considered more relevant in disease status classification. The VIP analysis identified IDL cholesterol concentration as the lipoprotein presenting the most relevant alterations between groups (Fig. 3a). Conversely, the score plot of the PLS-DA analysis clearly distinguished between pre- and post-surgery values because the components had a very slight overlap. The VIP analysis identified LMWM1 area as the parameter showing the most relevant pre-surgery and post-surgery differences (Fig. 3b).

## Discussion

Despite the conventional lipid panel not showing any significant differences between groups, we did find significant changes in the 1H-NMR-analyzed lipoprotein profile in patients with morbid obesity and NASH, when compared to patients without NASH. The key findings are that NASH was associated with a greater amount of total VLDL particles due to an increase in medium and small VLDL, and this change was related to higher concentrations of VLDL cholesterol. These alterations were more pronounced in patients with type 2 diabetes mellitus. In addition, patients with the most severe degrees of fibrosis had a greater amount of all subclasses of LDL particles. At present, there is a paucity of studies evaluating changes in lipoprotein metabolism in patients with liver disease, using 1H-NMR methods. For example, Siddiqui et al*.*^7^ did not find any significant differences in the lipoprotein profile between NASH and simple steatosis in non-diabetic patients with a moderate degree of obesity. Conversely, Männistö et al*.*^25^ observed an increase in VLDL and LDL concentrations in patients with morbid obesity and NASH. Overall and considering the variety of populations studied the results obtained suggest that alterations in the lipoprotein profile are aggravated as the patient's clinical severity increases in terms of hepatic alterations, the degree of obesity, or the presence of type 2 diabetes mellitus. Our study showed, as well, that patients with advanced fibrosis had the greatest alterations in the lipoprotein profile. A recent study has related fibrosis and lipoprotein metabolism via a receptor termed *Recepteur d’Origine Nantais* (Ron)^26^. This is a MET proto-oncogene receptor tyrosine kinase, the deficiency of which in mice results in the increase in concentrations of circulating VLDL and LDL, upregulation of collagen synthesis, and downregulation of matrix metallopeptidase-9 (an enzyme with collagenase activity). The possibility that there is a decreased activity of Ron in patients with NASH and fibrosis is an interesting hypothesis, and one that deserves further research.

There is a dearth of information regarding changes in glycoprotein values in patients with morbid obesity and liver disease. We did not find any significant association between these parameters and the presence of NASH, but we did find an increase in the areas of Glyc-A (which is an estimation of the protein-bound N-acetylglucosamine and *N*-acetylgalactosamine) and Glyc-F (which is an estimation of *N*-acetylglucosamine, *N*-acetylgalactosamine and *N*-acetylneuraminic acid not bound to proteins) in patients with diabetes. These latter results would be expected since protein glycosylation is an essential feature of diabetes. High Glyc-A and Glyc-B levels have been found in diabetes^10^, cardiovascular disease^27,28^, and other inflammatory diseases^11,29^. N-glycosylation of proteins can be a useful biomarker for the diagnosis of type 2 diabetes mellitus as well as for the identification and prognosis in groups at high risk of future diabetes^30,31^. If N-glycosylated proteins are confirmed as biomarkers, the results of the present study would add valuable information since our results indicate that N-glycoprotein levels are not altered with the degree of liver injury and, as such, do not constitute a confounding variable in the associated diabetes. ^1^H-NMR glycoprotein analysis adds further details in the detection albeit with low sensitivity and specificity of the levels of some soluble metabolites not directly related to glycosylation. In the present study we found an association between the LMWM 1 and 2 areas and the presence of NASH. These areas correspond, mainly, to glutamate and glutamine. These results are consistent with targeted metabolomics studies conducted in our research group in which high concentrations of these metabolites were noted in patients with morbid obesity and liver disease^19,32^.

The treatment-of-choice for morbid obesity is bariatric surgery. There are several techniques in performing this type of surgery, but the most commonly used are LSG and Roux-en-Y gastric bypass. To the best of our knowledge, the effects of LSG on ^1^H-NMR lipoprotein and glycoprotein profiles have not been reported in the literature, to date. The present study showed that one year after LSG, the patients had a significant decrease in the concentration of VLDL particles of all sizes, a decrease in VLDL cholesterol and triglyceride concentrations as well as Glyc-A, B, and F, and LMWM 1 and 2 areas. This was accompanied by an increase in the concentration of LDL particles of all sizes, in LDL particle diameter, in LDL cholesterol concentrations, and in small HDL particles and HDL cholesterol concentrations. These results differ from those obtained by Männistö et al*.*^25^ in patients with morbid obesity treated with Roux-en-Y gastric bypass; the authors reported a normalization of VLDL, IDL, and LDL levels and an increase in HDL one year after surgery. However, our results are similar to those of a study in patients with mild obesity receiving pharmacological treatment to induce NASH remission^33^. The authors reported that patients with NASH had high concentrations of small LDL and large VLDL subfractions, and that treatment was associated with an increase in LDL particle diameter and a decrease in VLDL concentrations. We are not sure of the cause of the increase in the concentration of LDL particles in our patients post-LSG, nor of the clinical consequences with respect to the long-term cardiovascular risk that this phenomenon may have. The increase in LDL concentrations together with the decrease in VLDL concentrations suggests a normalization of VLDL metabolism. This may be related to the improvement of insulin resistance and the normalization of liver function with weight loss^34^. Another possibility is an increase in the direct secretion of LDL by the liver. That the increase in LDL particles occurs, essentially, at the expense of large and medium-sized particles (i.e. those with less atherogenic capacity than the smaller particles), and with no increase in LDL cholesterol concentrations suggests that these changes are not necessarily detrimental to the patient’s cardiovascular disease status. Monitoring these patients for longer periods would clarify whether these changes in lipoproteins (concentrations and/or composition) persist and what effect they would have on the patient’s cardiovascular status.

In conclusion, our study reveals important alterations in the characteristics of the different classes of lipoproteins and glycoproteins in patients with obesity, especially in those with definite NASH and/or diabetes. It also indicates that most of these abnormalities can be reversed with LSG.

## Supplementary Information

Supplementary Information.

## References

[1] Ogden, C.L., Carroll, M.D., Kit, B.K. & Flegal, K.M. Prevalence of obesity among adults: United States, 2011–2012. *NCHS Data Brief.* 1–8 (2013). 24152742

[2] Joven J, Micol V, Segura-Carretero A, Alonso-Villaverde C, Menendez JA (2013). Polyphenols and the modulation of gene expression pathways: can we eat our way out of the danger of chronic disease?. Crit. Rev. Food Sci. Nutr..

[3] Cabré N (2019). Laparoscopic sleeve gastrectomy reverses non-alcoholic fatty liver disease modulating oxidative stress and inflammation. Metabolism..

[4] Kotronen A, Yki-Järvinen H (2008). Fatty liver: A novel component of the metabolic syndrome. Arterioscler. Thromb. Vasc. Biol..

[5] Fon Tacer K, Rozman D (2011). Nonalcoholic fatty liver disease: focus on lipoprotein and lipid deregulation. J. Lipids..

[6] Hamaguchi M (2007). Nonalcoholic fatty liver disease is a novel predictor cardiovascular disease. World J. Gastroenterol..

[7] Siddiqui MS (2015). Severity of nonalcoholic fatty liver disease and progression to cirrhosis are associated with atherogenic lipoprotein profile. Clin. Gastroenterol. Hepatol..

[8] Amor AJ (2017). Relationship between noninvasive scores of nonalcoholic fatty liver disease and nuclear magnetic resonance lipoprotein abnormalities: A focus on atherogenic dyslipidemia. J. Clin. Lipidol..

[9] Mallol R, Rodriguez MA, Brezmes J, Masana L, Correig X (2013). Human serum/plasma lipoprotein analysis by NMR: Application to the study of diabetic dyslipidemia. Prog. Nucl. Magn. Reson. Spectrosc..

[10] Lorenzo C, Festa A, Hanley AJ, Rewers MJ, Escalante A, Haffner SM (2017). Novel protein glycan-derived markers of systemic inflammation and C-reactive protein in relation to glycemia, insulin resistance, and insulin decretion. Diabetes Care.

[11] Fuertes-Martín R (2019). Glycoprotein A and B height-to-width ratios as obesity-independent novel biomarkers of low-grade chronic inflammation in women with Polycystic Ovary Syndrome (PCOS). J. Proteome Res..

[12] Surve A (2018). Does the future of laparoscopic sleeve gastrectomy lie in the outpatient surgery center? A retrospective study of the safety of 3162 outpatient sleeve gastrectomies. Surg. Obes. Relat. Dis..

[13] Taitano AA, Markow M, Finan JE, Wheeler DE, Gonzalvo JP, Murr MM (2015). Bariatric surgery improves histological features of nonalcoholic fatty liver disease and liver fibrosis. J. Gastrointest. Surg..

[14] Lassailly G (2015). Bariatric surgery reduces features of nonalcoholic steatohepatitis in morbidly obese patients. Gastroenterology.

[15] Luo RB (2018). How bariatric surgery affects liver volume and fat density in NAFLD patients. Surg. Endosc..

[16] von Schönfels W (2018). Histologic improvement of NAFLD in patients with obesity after bariatric surgery based on standardized NAS (NAFLD activity score). Surg. Obes. Relat. Dis..

[17] Ayloo S, Guss C, Pentakota SR, Hanna J, Molinari M (2019). Minimally invasive sleeve gastrectomy as a surgical treatment for nonalcoholic fatty liver disease in liver transplant recipients. Transplant. Proc..

[18] Ganguli S, DeLeeuw P, Satapathy SK (2019). A review of current and upcoming treatment modalities in non-alcoholic fatty liver disease and non-alcoholic steatohepatitis. Hepat. Med..

[19] Cabré N (2020). Plasma metabolic alterations in patients with severe obesity and non-alcoholic steatohepatitis. Aliment. Pharmacol. Ther..

[20] Mechanick JI (2013). Clinical practice guidelines for the perioperative nutritional, metabolic, and nonsurgical support of the bariatric surgery patient–2013 update: Cosponsored by American Association of Clinical Endocrinologists, the Obesity Society, and American Society for Metabolic & Bariatric Surgery. Surg. Obes. Relat. Dis..

[21] Vives M (2017). Analysis of gastric physiology after laparoscopic sleeve gastrectomy (LSG) with or without antral preservation in relation to metabolic response: A randomised study. Obes. Surg..

[22] Bedossa P (2012). Histopathological algorithm and scoring system for evaluation of liver lesions in morbidly obese patients. Hepatology.

[23] Kleiner DE (2005). Design and validation of a histological scoring system for nonalcoholic fatty liver disease. Hepatology.

[24] Chong J, Wishart DS, Xia J (2019). Using MetaboAnalyst 4.0 for comprehensive and integrative metabolomics data analysis. Curr. Protoc. Bioinform..

[25] Männistö VT (2014). Lipoprotein subclass metabolism in nonalcoholic steatohepatitis. J. Lipid Res..

[26] Allen J, Zhang J, Quickel MD, Kennett M, Patterson AD, Hankey-Giblin PA (2018). Ron receptor signaling ameliorates hepatic fibrosis in a diet-induced nonalcoholic steatohepatitis mouse model. J. Proteome Res..

[27] Otvos JD (2018). Relations of GlycA and lipoprotein particle subspecies with cardiovascular events and mortality: A post hoc analysis of the AIM-HIGH trial. J. Clin. Lipidol..

[28] Muhlestein JB (2018). GlycA and hsCRP are independent and additive predictors of future cardiovascular events among patients undergoing angiography: The intermountain heart collaborative study. Am. Heart J..

[29] Fuertes-Martín R (2018). Characterization of (1)H NMR plasma glycoproteins as a new strategy to identify inflammatory patterns in rheumatoid arthritis. J. Proteome Res..

[30] Rudman N, Gornik O, Lauc G (2019). Altered N-glycosylation profiles as potential biomarkers and drug targets in diabetes. FEBS Lett..

[31] Dotz V, Wuhrer M (2019). N-glycome signatures in human plasma: associations with physiology and major diseases. FEBS Lett..

[32] Rodríguez-Gallego E (2015). Mapping of the circulating metabolome reveals α-ketoglutarate as a predictor of morbid obesity-associated non-alcoholic fatty liver disease. Int. J. Obes. (Lond).

[33] Corey KE (2019). Relationship between resolution of non-alcoholic steatohepatitis and changes in lipoprotein sub-fractions: a post-hoc analysis of the PIVENS trial. Aliment. Pharmacol. Ther..

[34] Jiang ZG, Robson SC, Yao Z (2013). Lipoprotein metabolism in nonalcoholic fatty liver disease. J. Biomed. Res..

