# Supplementary material for: Laparoscopic sleeve gastrectomy alters 1H-NMR-measured lipoprotein and glycoprotein profile in patients with severe obesity and nonalcoholic fatty liver disease
Source: Sci Rep. 2021 Jan 14;11:1343. doi: 10.1038/s41598-020-79485-7 (PMC7809416; doi:10.1038/s41598-020-79485-7)
Supplement: Supplementary file 1 — Supplementary Information. [file 41598_2020_79485_MOESM1_ESM.doc]

**Supplementary materials corresponding to:**

**Laparoscopic sleeve gastrectomy alters ^1^H-NMR-measured lipoprotein and glycoprotein profile in patients with severe obesity and non-alcoholic fatty liver disease**

**Noemí Cabré^1,2^, Míriam Gil^3^, Núria Amigó^3^, Fedra Luciano-Mateo^1,2^, Gerard Baiges-Gayà^1,2^, Salvador Fernández-Arroyo^1,2^, Elisabet Rodríguez-Tomàs^1,2^, Anna Hernández-Aguilera^1,2^, Helena Castañé^1,2^, Marta París^4^, Fàtima Sabench^4^, Daniel Del Castillo^4^, Jordi Camps^1,2^, Jorge Joven^1,2,5^**

**^1^ Universitat Rovira i Virgili, Department of Medicine and Surgery, C. Sant Llorenç, 21, 43201 Reus, Spain. ^2^ Unitat de Recerca Biomèdica (URB-CRB), Hospital Universitari de Sant Joan, Institut d’Investigacio Sanitaria Pere Virgili, Universitat Rovira i Virgili, C. Sant Joan s/n, 43201 Reus, Spain. ^3^ Biosfer Teslab, Universitat Rovira i Virgili, Av. Universitat 1, 43204 Reus, Spain. ^4^ Department of Surgery, Hospital Universitari de Sant Joan, Institut d’Investigació Sanitaria Pere Virgili, Universitat Rovira i Virgili, Av. Doctor Josep Laporte 2, 43204 Reus, Spain. ^5^ The Campus of International Excellence Southern Catalonia, Tarragona, Spain.**

Correspondence and requests for materials should be addressed to J.C. (email: [jcamps@grupsagessa.com](mailto:jcamps@grupsagessa.com)) or J.J. (email: [jjoven@grupsagessa.comjjoven@grupsagessa.com](mailto:jjoven@grupsagessa.com)).

Supplementary Table 1

Clinical, biochemical, and histological variables segregated with respect to the degree of hepatic steatosis, lobular inflammation and fibrosis

| Steatosis | <5%  (n = 32) | 6-33%  (n = 58) | 34-66  (n = 42) | >67%  (n =22) | *P* value |
| --- | --- | --- | --- | --- | --- |
| Female, n (%) | 25 (78.1) | 42 (72.4) | 36 (85.7) | 15 (68.2) | 0.333 |
| Age, years | 50 (40-59) | 51 (43-61) | 56 (47-63) | 46 (42-54)^e^ | 0.155 |
| BMI, Kg/m^2^ | 47.0 (44.0-51.6) | 49.4 (44.7-53.5) | 48.1 (44.7-54.0) | 49.9 (43.6-55.5) | 0.776 |
| Glucose, mmol/L | 6.8 (6.2-8.3) | 7.3 (5.6-8.5) | 8.5 (7.1-11.5)^a,c^ | 8.4 (6.3-10.5) | 0.018 |
| Insulin, pmol/L | 107.3 (55.9-167.3) | 61.1 (35.9-137.1) | 96.5 (63.5-141.3) | 124.3 (76.3-180.9) | 0.197 |
| HOMA-IR | 4.3 (2.4-7.4) | 3.0 (1.4-6.9) | 5.5 (3.2-9.9)^c^ | 6.2 /3.7-9.6)^d^ | 0.038 |
| AST, U/L | 24 (18-54) | 30 (18-36) | 54 (30-78)^a,c^ | 60 (36-90)^b,d^ | <0.001 |
| ALT, U/L | 30 (18-54) | 30 (18-36) | 54 (30-72)^a,c^ | 54 (36-96)^b,d^ | <0.001 |
| GGT, U/L | 24 (18-36) | 24 (12-30) | 30 (18-54)^c^ | 36 (25-90)^b,d^ | <0.001 |
| Lobular Inflammation | No foci  (n = 23) | <2 per 200x field  (n = 57) | 2-4 per 200x field  (n = 54) | >4 per 200x field  (n = 20) | *P* value |
| Female, n (%) | 18 (78.2) | 44 (77.2) | 39 (72.2) | 16 (80.0) | 0.792 |
| Age, years | 50 (40-63) | 51 (43-62) | 53 (44-60) | 52 (43-61) | 0.977 |
| BMI, Kg/m^2^ | 46.6 (42.9-53.3) | 48.2 (43.5-53.7) | 49.7 (44.6-54.0) | 49.0 (44.7-53.9) | 0.678 |
| Glucose, mmol/L | 7.6 (6.5-9.0) | 7.9 (6.4-9.9) | 7.5 (5.6-8.8) | 7.4 (6.5-11.2) | 0.668 |
| Insulin, pmol/L | 89.5 (38.5-147.2) | 94.8 (42.8-142.3) | 83.3 (49.6-150.7) | 99.0 (59.7-168.7) | 0.949 |
| HOMA-IR | 4.6 (1.9-7.9) | 4.5 (1.9-8.1) | 4.1 (2.4-7.3) | 6.5 (2.5-8.9) | 0.838 |
| AST, U/L | 36 (24-54) | 30 (18-60) | 36 (24-60) | 36 (30-54) | 0.920 |
| ALT, U/L | 30 (18-54) | 36 (24-60) | 36 (34-48) | 36 (30-54) | 0.931 |
| GGT, U/L | 24 (12-30) | 24 (18-42) | 24 (12-42) | 24 (18-54) | 0.581 |
| Fibrosis stage | Stage 0  (n = 41) | Stage 1  (n = 53) | Stage 2  (n = 40) | Stage 3  (n = 20) | *P* value |
| Female, n (%) | 30 (73.1) | 41 (77.4) | 29 (72.5) | 15 (75.0) | 0.916 |
| Age, years | 52 (42-63) | 52 (42-61) | 50 (44-60) | 52 (45-57) | 0.968 |
| BMI, Kg/m^2^ | 47.3 (42.9-51.3) | 48.6 (43.5-54.7) | 49.5 (45.2-55.7) | 49.2 (44.5-53.4) | 0.304 |
| Glucose, mmol/L | 7.9 (6.4-9.4) | 7.3 (6.5-9.0) | 7.5 (5.5-9.8) | 8.4 (5.7-10.3) | 0.721 |
| Insulin, pmol/L | 96.5 (55.9-178.1) | 96.8 (40.4-170.8) | 72.5 (44.7-129.6) | 95.8 (59.9-140.6) | 0.400 |
| HOMA-IR | 5.1 (2.6-8.2) | 5.0 (1.8-10.2) | 3.9 (1.9-6.1) | 5.2 (3.0-9.5) | 0.365 |
| AST, µkat/L | 30 (24-42) | 30 (24-48) | 36 (18-60) | 48 (30-78)^f,g^ | 0.041 |
| ALT, µkat/L | 30 (24-48) | 30 (24-42) | 36 (18-66) | 48 (30-72)^f,g^ | 0.047 |
| GGT, µkat/L | 24 (12-36) | 18 (12-30) | 24 (18-66) | 36 (24-54)^f,g^ | 0.009 |

Values are shown as number of cases and percentages, or medians and interquartile ranges in parentheses. ALT: alanine aminotransferase; AST: aspartate aminotransferase; BMI: body mass index; GGT: γ-glytamyl transferase; HOMA-IR: homeostatic model assessment of insulin resistance. Statistical analyses were performed with the Kruskal-Wallis one-way analysis of variance for continuous variables and χ-square test for categorical variables. Superscript letters indicate significant (at least p ≤ 0.05) differences between: ^a^ <5% *vs.* 34-66%, ^b^ <5% *vs.* >67%, ^c^ 6-33% *vs.* 34-66%, ^d^6-33% *vs.* >67%, ^e^34-66% *vs.*>67%, ^f^ Stage 0 *vs.* Stage 3 and ^g^Stage 1 *vs.* Stage 3. Data collected at the time of surgery.

Supplementary Table 2

^1^H-NMR analysis of serum cholesterol and triglyceride concentrations in lipoprotein fractions, lipoprotein particle diameter and glycoprotein variables segregated with respect to the degree of hepatic steatosis

| Steatosis | <5%  (n=32) | 6-33%  (n=58) | 34-66  (n=42) | >67%  (n=22) | *P* value |
| --- | --- | --- | --- | --- | --- |
| Cholesterol, mg/dL |  |  |  |  |  |
| VLDL | 18.0 (14.-136.4)-24.8) | 23.0 (17.2-28.5)^a^ | 26.9 (17.6-35.6)^b^ | 31.9 (18.4-44.3)^c^ | 0.011 |
| IDL | 9.4 (7.2-10.7) | 10.2 (8.1-12.2) | 11.4 (9.3-15.4)^b,d^ | 15.1 (10.6-17.3)^c,e,f^ | <0.001 |
| LDL | 108.4 (90.0-136.4) | 107.8 (93.3-119.6) | 104.0 (88.4-124.7) | 102.8 (76.5-122.7) | 0.674 |
| HDL | 45.7 (40.9-52.0) | 47.3 (37.7-53.6) | 44.3 (39.4-51.6) | 43.0 (39.3-51.3) | 0.856 |
| Triglycerides, mg/dL |  |  |  |  |  |
| VLDL | 59.8 (52.1-89.7) | 82.4 (60.1-112.6)^a^ | 96.8 (60.6-126.4)^b^ | 98.3 (59.0-153.9)^c^ | 0.009 |
| IDL | 10.4 (8.5-11.0) | 10.9 (9.3-12.9)^a^ | 12.2 (10.2-15.5)^b^ | 15.6 (11.2-17.7)^c,e^ | <0.001 |
| LDL | 14.1 (11.6-17.1) | 15.0 (12.4-17.8) | 16.1 (12.8-21.2) | 21.8 (12.4-23.9)^c,e^ | 0.016 |
| HDL | 18.7 (13.0-24.6) | 19.4 (15.8-25.9) | 20.6 (17.2-29.7)^b^ | 22.2 (18.7-39.2)^c,e^ | 0.013 |
| Lipoprotein particle diameter (Z) |  |  |  |  |  |
| VLDL | 42.1 (41.9-42.3) | 42.0 (41.8-42.3) | 42.0 (41.8-42.3) | 42.1 (41.9-42.3) | 0.312 |
| LDL | 21.0 (20.8-21.7) | 20.9 (20.5-21.0)^a^ | 20.9 (20.7-21.5)^b^ | 21.0 (20.9-21.2)^e^ | 0.020 |
| HDL | 8.3 (8.2-8.4) | 8.3 (8.2-8.4) | 8.3 (8.2-8.4) | 8.3 (8.2-8.4) | 0.865 |
| Glycoprotein variables |  |  |  |  |  |
| LMWM1 area | 0.27 (0.21-0.35) | 0.24 (0.16-0.35) | 0.27 (0.16-0.36) | 0.33 (0.24-0.48)^e,f^ | 0.116 |
| LMWM2 area | 1.9 (1.6-2.3) | 2.3 (1.7-2.9)^a^ | 2.4 (1.7-2.8)^b^ | 2.6 (2.2-3.3)^c^ | 0.013 |
| Glyc-A area | 5.7 (4.9-6.1) | 5.8 (5.1-6.8) | 5.7 (4.9-7.3) | 6.4 (5.1-7.3) | 0.239 |
| Glyc-B area | 2.2 (1.9-2.5) | 2.0 (1.8-2.5) | 2.2 (1.7-2.6) | 2.2-2.0-3.0) | 0.327 |
| Glyc-F area | 2.2 (2.0-2.5) | 2.4 (2.1-2.7) | 2.3 (1.8-2.7) | 2.6 (2.2-3.2)^c^ | 0.088 |
| Glyc-A width | 16.9 (16.5-17.5) | 17.3 (16.7-17.7) | 17.4 (16.9-17.9)^b^ | 17.3 (16.6-18.2)^c^ | 0.170 |
| Glyc-B width | 20.3 (19.1-21.2) | 19.6 (18.3-20.8) | 20.4 (18.8-22.1) | 20.1 (19.1-21.5)^f^ | 0.181 |
| Height/Width Glyc-A | 17.6 (15.-19.8) | 18.1 (16.6-20.9) | 17.6 (15.3-20.6) | 19.8 (17.0-22.5) | 0.179 |
| Height/Width Glyc-B | 5.0 (4.4-5.5) | 5.0 (4.6-5.8) | 4.9 (4.2-5.5) | 5.6 (4.8-6.4) | 0.159 |

Values are shown as medians and interquartile ranges in parentheses. HDL: high-density lipoproteins; IDL: intermediate-density lipoproteins; LMWM: low molecular weight molecules; LDL: low-density lipoproteins; VLDL: very-low density lipoproteins: Statistical analyses were performed using the Kruskal-Wallis one-way analysis of variance. Superscript letters indicate significant (at least p ≤ 0.05) differences: ^a^ <5% *vs 6-33%.* ^b^ <5% *vs.* 34-66%, ^c^ <5% *vs.* >67%, ^d^ 6-33% *vs.* 34-66%, ^e^ 6-33% *vs.* >67% and ^f^ 34-66% *vs.*>67%. Data collected at the time of surgery.

Supplementary Table 3

^1^H-NMR analysis of serum cholesterol and triglyceride concentrations in lipoprotein fractions, lipoprotein particle diameter and glycoprotein variables segregated with respect to the degree of fibrosis

|  | Stage 0  (n=41) | Stage 1  (n=53) | Stage 2  (n=40) | Stage 3  (n=20) | *P* value |
| --- | --- | --- | --- | --- | --- |
| Cholesterol, mg/dL |  |  |  |  |  |
| VLDL | 20.6 (16.0-32.5) | 21.8 (15.3-33.3) | 23.0 (17.0-30.9) | 29.7 (20.5-36.5)^d^ | 0.295 |
| IDL | 9.5 (7.6-13.7) | 10.5 (8.4-12.8) | 10.9 (9.2-13.4) | 12.8 (10.4-17.0)^b,c^ | 0.016 |
| LDL | 106.2 (85.7-117.4) | 101.7 (90.1-124.1) | 106.6 (92.8-124.1) | 120.8 (102.5-161.4)^b,c^ | 0.015 |
| HDL | 48.0 (39.1-57.1) | 46.9 (40.6-53.9) | 43.7 (38.2-48.8)^a^ | 44.1 (39.8-48.8) | 0.036 |
| Triglycerides, mg/dL |  |  |  |  |  |
| VLDL | 78.9 (56.4-125.4) | 73.9 (54.0-114.7) | 84.4 (61.2-101.6) | 104.8 (64.5-128.3) | 0.426 |
| IDL | 10.3 (8.7-14.6) | 11.0 (9.5-13.4) | 11.8 (9.6-12.9) | 13.1 (11.1-16.0)^b,c,d^ | 0.080 |
| LDL | 15.0 (11.7-19.7) | 15.8 (11.5-19.2) | 14.9 (12.5-19.3) | 20.2 (15.2-22.9)^b,c,d^ | 0.019 |
| HDL | 19.7 (17.5-27.3) | 20.1 (16.6-30.0) | 18.8 (15.2-25.3) | 21.1 (17.1-29.1) | 0.531 |
| Lipoprotein particle diameter (Z) |  |  |  |  |  |
| VLDL | 42.1 (41.9-42.3) | 42.0 (41.8-42.3) | 42.0 (41.8-42.3) | 42.0 (41.7-42.3) | 0.713 |
| LDL | 21.0 (20.7-21.1) | 20.9 (20.6-21.1) | 20.9 (20.7-21.1) | 21.1 (20.8-21.2) | 0.405 |
| HDL | 8.3 (8.2-8.4) | 8.3 (8.2-8.4) | 8.3 (8.2-8.4) | 8.3 (8.2-8.4) | 0.607 |
| Glycoprotein variables |  |  |  |  |  |
| LMWM1 area | 0.27 (0.17-0.37) | 0.28 (0.17-0.40) | 0.24 (0.11-0.31) | 0.31 (0.25-0.40)^d^ | 0.033 |
| LMWM2 area | 2.2 (1.8-2.8) | 2.2 (1.6-2.9) | 2.2 (1.8-2.7) | 2.7 (1.7-3.2) | 0.454 |
| Glyc-A area | 5.8 (4.9-7.1) | 5.7 (4.9-6.7) | 5.8 (5.1-6.3) | 6.6 (5.7-7.3)^c,d^ | 0.166 |
| Glyc-B area | 2.2 (1.8-2.7) | 2.1 (1.8-2.6) | 2.1 (1.8-2.6) | 2.2 (1.9-2.7) | 0.870 |
| Glyc-F area | 2.3 (2.1-2.8) | 2.4 (1.9-2.7) | 2.3 (2.0-2.6) | 2.7 (2.5-3.1)^b,c,d^ | 0.057 |
| Glyc-A width | 17.3 (16.6-17.9) | 17.2 (4.5-5.6) | 17.2 (16.6-17.6) | 17.5 (16.9-18.0) | 0.405 |
| Glyc-B width | 20.2 (18.8-21.5) | 20.0 (18.7-21.2) | 20.1 (18.5-21.4) | 19.7 (18.9-21.5) | 0.996 |
| Height/Width Glyc-A | 18.3 (16.1-21.6) | 17.5 (15.8-19.8) | 18.3 (15.8-20.3) | 20.3 (16.4-22.8)^c^ | 0.144 |
| Height/Width Glyc-B | 4.9 (4.5-6.1) | 5.0 (4.5-5.6) | 5.0 (4.4-5.5) | 5.4 (4.8-5.8) | 0.356 |

Values are shown as medians and interquartile ranges in parentheses. HDL: high-density lipoproteins; IDL: intermediate-density lipoproteins; LMWM: low molecular weight molecules; LDL: low-density lipoproteins; VLDL: very-low-density lipoproteins. Statistical analyses were performed using the Kruskal-Wallis one-way analysis of variance. Superscript letters indicate significant (at least p ≤ 0.05) differences: ^a^ Stage 0 *vs* Stage 2*;* ^b^ Stage 0 *vs.* Stage 3; ^c^ Stage 1 *vs.* Stage 3; ^d^ Stage 2 *vs.* Stage 3. Data collected at the time of surgery.

Supplementary Table 4

^1^H-NMR analysis of serum cholesterol and triglyceride concentrations in lipoprotein fractions, lipoprotein particle diameter and glycoprotein variables segregated with respect to the degree of lobular inflammation

|  | No foci  (n=23) | <2 per 200x field  (n=57) | 2-4 per 200x field  (n=54) | >4 per 200x field  (n=21) | *P* value |
| --- | --- | --- | --- | --- | --- |
| Cholesterol, mg/dL |  |  |  |  |  |
| VLDL | 17.9 (15.9-27.4) | 23.2 (15.4-35.8) | 22.4 (17.9-30.1) | 24.6 (16.8-34.9) | 0.646 |
| IDL | 9.4 (7.7-11.0) | 10.7 (8.7-14.4) | 11.0 (9.3-13.7)^a^ | 10.5 (8.1-14.3) | 0.237 |
| LDL | 104.3 (91.7-126.6) | 109.3 (89.9-126.0) | 106.5 (91.4-121.4) | 103.6 (86.7-119.1) | 0.956 |
| HDL | 44.1 (38.5-53.9) | 47.8 (38.0-55.1) | 44.7 (38.6-51.0) | 44.5 (40.9-52.5) | 0.530 |
| Triglycerides, mg/dL |  |  |  |  |  |
| VLDL | 75.4 (57.5-101.3) | 88.7 (53.8-127.8) | 79.1 (57.0-114.4) | 89.0 (59.7-109.8) | 0.934 |
| IDL | 9.8 (8.6-12.2) | 11.4 (9.3-15.0) | 11.1 (9.7-14.6) | 11.4 (10.1-14.2) | 0.312 |
| LDL | 14.2 (10.5-16.5) | 15.3 (12.8-21.1) | 16.4 (11.9-19.7) | 15.9 (12.8-21.6) | 0.295 |
| HDL | 19.2 (14.7-22.1) | 19.4 (15.5-29.5) | 19.3 (15.8-25.8) | 24.3 (19.3-30.7)^b,c^ | 0.093 |
| Lipoprotein particle diameter (Z) |  |  |  |  |  |
| VLDL | 41.9 (41.8-42.3) | 42.0 (41.8-42.3) | 42.0 (41.8-42.5) | 42.2 (42.0-42.4)^b,c^ | 0.111 |
| LDL | 20.9 (20.4-21.1) | 21.0 (20.7-21.1) | 20.9 (20.7-21.1) | 21.0 (20.8-21.2)^b,c^ | 0.108 |
| HDL | 8.3 (8.2-8.3) | 8.3 (8.2-8.4) | 8.3 (8.2-8.4) | 8.3 (8.2-8.4) | 0.677 |
| Glycoprotein variables |  |  |  |  |  |
| LMWM1 area | 0.23 (0.15-0.32) | 0.29 (0.21-0.40) | 0.25 (0.14-0.36) | 0.27 (0.20-0.35) | 0.260 |
| LMWM2 area | 2.0 (1.5-2.7) | 2.3 (1.6-2.9) | 2.2 (1.7-2.7) | 2.2 (1.7-2.9) | 0.764 |
| Glyc-A area | 5.7 (4.7-6.7) | 5.8 (5.1-7.2) | 5.7 (4.9-7.0) | 5.9 (5.1-6.3) | 0.829 |
| Glyc-B area | 2.2 (1.6-2.5) | 2.3 (1.9-2.6) | 2.0 (1.8-2.7) | 2.1 (1.8-2.5) | 0.548 |
| Glyc-F area | 2.3 (1.9-2.8) | 2.4 (2.1-2.8) | 2.4 (2.0-2.8) | 2.5 (2.1-2.7) | 0.896 |
| Glyc-A width | 17.2 (16.8-17.7) | 17.4 (16.9-17.8) | 17.1 (16.6-17.6) | 17.4 (16.8-17.8) | 0.544 |
| Glyc-B width | 19.9 (18.7-21.0) | 20.0 (18.8-22.0) | 20.1 (18.8-20.8) | 20.0 (18.7-20.7) | 0.847 |
| Height/Width Glyc-A | 18.0 (15.9-20.7) | 18.2 (16.3-20.7) | 17.9 (15.6-21.1) | 18.5 (15.9-19.9) | 0.958 |
| Height/Width Glyc-B | 5.1 (4.4-5.8) | 5.1 (4.5-5.7) | 4.9 (4.4-5.8) | 5.1 (4.6-5.6) | 0.988 |

Values are shown as medians and interquartile ranges in parentheses. HDL: high-density lipoproteins; IDL: intermediate- density lipoproteins; LMWM: low molecular weight molecules; LDL: low-density lipoproteins; VLDL: very-low-density lipoproteins: Statistical analyses were performed using the Kruskal-Wallis one-way analysis of variance. Superscript letters indicate significant (at least p ≤ 0.05) differences: ^a^ No foci *vs.* 2-4 per 200x field; ^b^ No foci *vs.* >4 per 200x field; ^c^ 2-4 per 200x field *vs* >4 per 200x field. Data collected at the time of surgery.

Supplementary Table 5

^1^H-NMR analysis of serum cholesterol and triglyceride concentrations in lipoprotein fractions, lipoprotein particle diameter and glycoprotein variables segregated with respect to the presence of type 2 diabetes mellitus

|  | Non-Diabetes  (n=84) | Diabetes*  (n=70) | *P* value |
| --- | --- | --- | --- |
| Cholesterol, mg/dL |  |  |  |
| VLDL | 20.5 (15.3-27.8) | 26.2 (18.0-38.6) | 0.002 |
| IDL | 10.3 (8.2-11.9) | 11.5 (9.0-16.0) | 0.011 |
| LDL | 108.9 (93.0-125.5) | 103.6 (85.3-121.4) | 0.156 |
| HDL | 46.5 (39.7-51.5) | 44.1 (38.5-52.7) | 0.730 |
| Triglycerides, mg/dL |  |  |  |
| VLDL | 71.6 (53.5-97.3) | 95.5 (66.0-134.0) | <0.001 |
| IDL | 10.8 (9.0-12.7) | 12.3 (10.1-16.2) | 0.002 |
| LDL | 15.6 (12.2-17.9) | 15.5 (12.7-21.8) | 0.180 |
| HDL | 18.8 (15.4-25.4) | 21.9 (18.6-30.8) | 0.002 |
| Lipoprotein particle diameter (Z) |  |  |  |
| VLDL | 42.0 (41.9-42.3) | 42.0 (41.7-42.2) | 0.101 |
| LDL | 21.0 (20.8-21.1) | 20.9 (20.6-21.1) | 0.097 |
| HDL | 8.3 (8.2-8.4) | 8.3 (8.2-8.4) | 0.553 |
| Glycoprotein variables |  |  |  |
| LMWM1 area | 0.27 (0.16-0.35) | 0.28 (0.18-0.40) | 0.582 |
| LMWM2 area | 2.1 (1.7-2.6) | 2.5 (1.7-3.0) | 0.050 |
| Glyc-A area | 5.7 (5.0-6.5) | 6.1 85.0-7.4) | 0.032 |
| Glyc-B area | 2.1 (1.8-2.5) | 2.2 (1.8-2.7) | 0.213 |
| Glyc-F area | 2.3 (2.0-2.6) | 2.5 (2.1-3.0) | 0.038 |
| Glyc-A width | 17.2 (16.6-17.6) | 17.3 (16.9-18.0) | 0.122 |
| Glyc-B width | 20.0 (18.8-21.1) | 20.1 (18.7-21.6) | 0.794 |
| Height/Width Glyc-A | 17.6 (15.7-20.5) | 18.5 (16.3-21.3) | 0.169 |
| Height/Width Glyc-B | 4.9 (4.4-5.7) | 5.1 (4.6-5.8) | 0.185 |

Values are shown as medians and interquartile range (in parentheses). HDL: high-density lipoproteins; IDL: intermediate-density lipoproteins; LDL: low-density lipoproteins; P: particle number; VLDL: very-low-density lipoproteins; Z: particle diameter. Statistical analyses were performed using the Wilcoxon rank-sum test. Data collected at the time of surgery. * Diabetic patients were diagnosed by following the criteria of the American Diabetes Association. These patients have been suffering from this disease for years, as it we verified before surgery that their morbid obesity is not due to any treatable endocrine disorder.
